# Generalized Mohr-Coulomb strain criterion for bulk metallic glasses under complex compressive loading

**DOI:** 10.1038/s41598-019-49085-1

**Published:** 2019-08-29

**Authors:** Li Yu, Tzu-Chiang Wang

**Affiliations:** 1 State Key Laboratory of Nonlinear Mechanics, Institute of Mechanics, Chinese Academy of Sciences, Beijing, 100190 China; 2 School of Engineering Sciences, University of Chinese Academy of Sciences, Beijing, 100049 China

**Keywords:** Mechanical engineering, Theory and computation

## Abstract

The Mohr-Coulomb (M-C) stress criterion is widely applied to describe the pressure sensitivity of bulk metallic glasses (BMGs). However, this criterion is incapable of predicting the variation in fracture angles under different loading modes. Moreover, the M-C criterion cannot describe the plastic fracture of BMGs under compressive loading because the nominal stress of most BMGs remains unchanged after the materials yield. Based on these limitations, we propose a new generalized M-C strain criterion and apply it to analyze the fracture behaviors of two typical Zr-based BMG round bar specimens under complex compressive loading. In this case, the predicted initial yielding stress is in good agreement with the experimental results. The theoretical results can also describe the critical shear strain and fracture angle of BMGs that are associated with the deformation mode.

## Introduction

Among current advanced materials, bulk metallic glasses (BMGs) have attracted wide attention and have great applications in functional and structural materials due to their outstanding mechanical, chemical, and physical properties^1–5^. In particular, metallic glasses have a high strength approaching the theoretical limit and have a uniquely high capacity to store elastic energy^6–10^. However, due to localized shear bands, the limited global plasticity of BMGs restricts the application of BMGs as a structural material. Thus, many studies in the last two decades were dedicated to overcoming this barrier and understanding the plasticity of BMGs. Many factors and ideas have been proposed to improve the ductility of BMGs, such as the BMG composition^11–14^, electrodeposition^15^, confining pressure^16,17^, and atomic scale effects^18^. Moreover, it is also important to investigate the fracture mechanism and fracture criteria of BMGs^19–21^ and to provide safe reference for their potential application. The existing fracture criteria, such as the traditional stress Mohr-Coulomb (M-C) criterion^22,23^, ellipse criterion^24^, and hyperbola criterion^25^, focus on describing the pressure sensitivity of BMGs^16,26–28^. The elastic-perfectly plastic behavior of BMGs under compressive loading is always ignored in these stress criteria because of the limited plasticity of previous BMGs^11,29,30^. However, the considerable plasticity of newly developed BMGs cannot be ignored; as the cross-sectional area increases under compressive loading, the nominal stress of BMGs remains unchanged, and the Cauchy stress decreases. This finding indicates that the stress criteria cannot accurately predict the failure behaviors of the newly developed BMGs. In addition, the existing criteria usually focus on fracture behavior under simple loading, but materials always suffer complex loading in engineering applications. Moreover, experiments under complex loading show that the macroscopic plasticity of BMGs will be enhanced with increasing superimposed confining pressure^16,17^. This finding indicates that the loading mode is important to the fracture behavior of BMGs. Therefore, it is necessary to establish a more suitable compression fracture criterion and a method to analyze the fracture behavior of BMGs under complex compressive loading.

**Figure 1:**
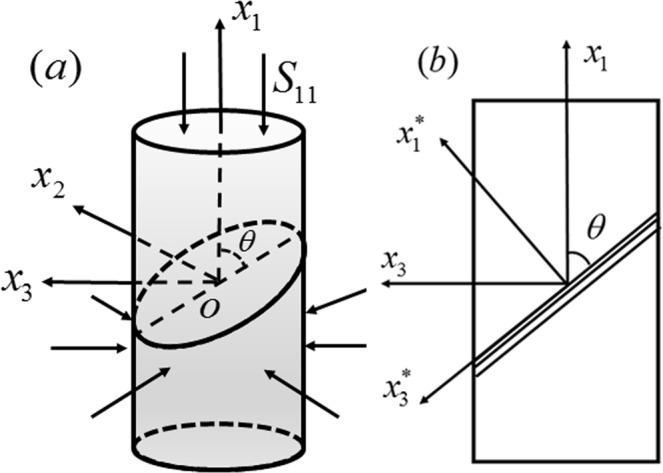
Graphical representation of the stress state of the round bar. (**a**) A round bar subjected to axial compression and a superimposed confining pressure, where *θ* represents the angle between the fracture plane and the *x*_1_axis. (**b**) Section diagram of the round bar.

Among the criteria introduced above, the M-C stress criterion is widely accepted and applied by scholars because of its simple form and clear physical meaning^16,19,21,31^. Therefore, we provide a new generalized M-C strain failure criterion for BMGs. By taking a round bar as the research object, the fracture behavior of BMGs under complex compressive loading will be discussed in this paper. According to the stress state shown in Fig. 1, we can assume that the normal of the fracture plane is located at the *x*_1_-*x*_3_ plane due to the symmetry of the round bar. The confining pressure is proportional to the axial stress: *S*_22_ = *S*_33_ = *ρS*_11_(*S*_11_ < 0), where *ρ* is the proportionality coefficient between the axial stress *S*_11_ and the confining pressure *S*_33_.

## Results

### Generalized Mohr-Coulomb strain failure criterion

To describe the fracture behaviors of BMGs under complex compressive loading, the M-C criterion should be improved due to the following aspects:

1. Compression experiments of round bar specimens of BMGs show that the axial nominal stress *S*_11_(as shown in Fig. 1(a)) always remains unchanged once the material yields^11,26,32^, correspondingly, the Cauchy stress decreases as the cross-sectional area increases. In this case, the relation between the stress state and the strain state of BMGs is no longer one-to-one once the material yields. This finding indicates that the strain state is more suitable than the stress state for predicting the fracture behaviors of BMGs.
2. The traditional M-C stress criterion is *τ* = *τ*_0_ + *μσ*, where *σ*and *τ* are the normal stress and shear stress on the fracture plane, respectively; *τ*_0_ is the pure shear strength; and *μ* is an important constant to measure the influence of normal stress, which is directly related to the frictional (fracture) angle *θ* (*μ* = tan*θ*). However, experiments show that the fractures in BMGs occur along different angles under different loading modes^21,31^. This finding means that the parameter *μ* in the traditional M-C criterion should not be a constant but should vary with the loading or deformation mode.
3. Due to the different production processes, there is a very large gap in the plasticity of different types of BMGs, which should be considered in the new criterion. Hence, to describe the fracture behaviors of BMGs, we propose a generalized M-C strain criterion that can be expressed as

1 $$\frac{\gamma }{2}+{\alpha }_{C}\varepsilon =\frac{{\gamma }_{Cf}}{2}\,,$$

where *ε* and *γ*/2 are the normal strain and the shear strain along the fracture plane, respectively, as shown in Fig. 1(b). The strain state (*ε*,*γ*/2) is related to the normal strain *ε*_1_ and *ε*_3_, which can be expressed as $\gamma /2=({\varepsilon }_{3}-{\varepsilon }_{1})\,\sin \,2\theta /2$ and $\varepsilon =({\varepsilon }_{3}+{\varepsilon }_{1})/2+({\varepsilon }_{3}-{\varepsilon }_{1})\cos \,2\theta /2$. Here, we define a parameter *ρ*′ = *ε*_3_/*ε*_1_ to describe the deformation state, and *α*_*C*_ = *α*_*C*0_(1 + *ρ*′) is a new form of intrinsic pressure-sensitivity parameter, which is related to the deformation mode. Note that *γ*_*Cf*_ is the critical shear strain, which describes the ability of materials to resist shear deformation and can be approximately expressed as

2 $${\gamma }_{Cf}={\gamma }_{C0}[1+\beta (1+\rho ^{\prime} )]$$

where *γ*_*C*0_ is the critical shear strain for *ρ*′ = −1 and *β* is a material constant. The generalized M-C strain criterion (Eq. (1)) describes the shear failure accompanied by the influence of pressure sensitivity. The fracture of BMGs occurs once the shear strain along the fracture plane reaches the critical value *γ*_*Cf*_.

The new generalized M-C strain criterion predicts the fracture behaviors of BMGs by the strain state along the fracture plane. However, the strain state along the fracture plane cannot be directly obtained in engineering applications. Thus, it is also important to predict the location of the fracture plane under different deformation modes. Our previous studies provide a universal formula that predicts the location of the most dangerous plane by seeking the tangent to the fracture line^33,34^. This formula can be rewritten as

3 $$tg(2\theta )=\frac{\partial F/\partial (\gamma /2)}{\partial F/\partial \varepsilon }.$$

*F*(*ε*,*γ*/2) is the fracture function, which can be expressed as

4 $$F=\frac{\gamma }{2}+{\alpha }_{C}\varepsilon -\frac{{\gamma }_{Cf}}{2}\,.$$

Substituting the above equation into Eq. (3), we can obtain the fracture angle for different deformation modes, which can be expressed as

5 $$ctg(2\theta )={\alpha }_{C0}(1+\rho ^{\prime} ).$$

**Figure 2:**
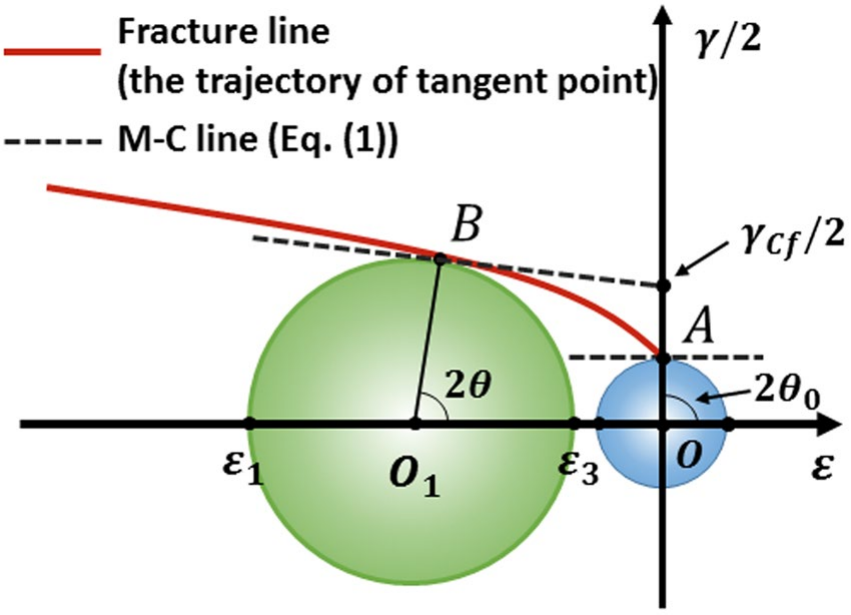
Graphical representation of the new strain criterion. Points A (pure shear strain) and B (general case) are the tangent points of the M-C line and Mohr’s circle. The red line represents the critical fracture locus of the new fracture criterion.

For the new criterion (Eq. (1)), the dependence between the shear strain *γ*/2 and the normal strain *ε* is no longer linear. Specifically, the material parameters in the traditional M-C stress criterion are independent of the loading mode, but these parameters are related to the deformation mode according to the current criterion. As illustrated in Fig. 2, two M-C lines (dashed lines) correspond to two different deformation modes (point A represents the pure shear state and point B represents one of the general cases). The fracture occurs at the time that the critical strain in the Mohr’s circle is tangent to the fracture line of the new fracture function F. The fracture angle and the fracture strain state can be obtained from the tangent point. Thus, the new generalized M-C strain criterion is different from the traditional M-C criterion. The new criterion does not directly indicate the fracture curve of BMGs but gives the critical tangent point in the Mohr’s circle for different deformation modes. The complete fracture line shown in Fig. 2 (red line) is the trajectory of the tangent point in different deformation modes.

### Yield criterion and constitutive relation

Once we know the strain state of a material, the new criterion can provide a reference for their safety. However, in many cases, we only know the stress state of a material but cannot directly obtain their strain state^16,26^. Thus, the corresponding constitutive relationship also needs to be discussed. In this paper, we choose the Drucker-Prager (D-P) criterion to describe the yield of BMGs because this criterion considers the influence of triaxial stress (pressure sensitivity) on material yielding. The nominal stress *S*_*ij*_ is used to replace Cauchy stress because the Cauchy stress decreases during deformation. The new form of the D-P criterion is

6 $$f={T}_{e}+\alpha {T}_{m}={f}_{0},$$

where ${T}_{e}=\sqrt{{T}_{ij}^{\text{'}}{T}_{ij}^{\text{'}}/2}$ is the effective stress, ${T}_{ij}^{\text{'}}={T}_{ij}-{T}_{m}{\delta }_{ij}$ is the deviatoric stress, *T*_*m*_ = *T*_*kk*_/3 is the mean stress, and the stress tensor *T*_*ij*_ = (*S*_*ij*_ + *S*_*ji*_)/2. The parameter *α* represents the pressure sensitivity of yielding, and the yield strength *f*_0_ represents the ability of materials to resist yielding under pure shear loading. Both *α*_*C*_ and *α* are pressure-sensitivity parameters, but they correspond to two different mechanical behaviors, fracture and yield, respectively. The plastic deformation of BMGs obeys the plastic normality rule, which indicates the direction of the plastic strain increment. Thus, the strain rate tensor *D*_*ij*_ can be written as

7 $${D}_{ij}=\frac{{\dot{T}}_{ij}^{\text{'}}}{2G}+\frac{{\dot{T}}_{m}}{K}{\delta }_{ij}+{\dot{\bar{\varepsilon }}}_{p}(\frac{{T}_{ij}^{\text{'}}}{2{T}_{e}}+\frac{1}{3}{\delta }_{ij}\alpha ),$$

where *G* and *K* are the elastic shear modulus and the bulk modulus, respectively (the specific derivations are shown in the Methods section).

### Fracture behaviors of Vit-105 BMGs

**Figure 3:**
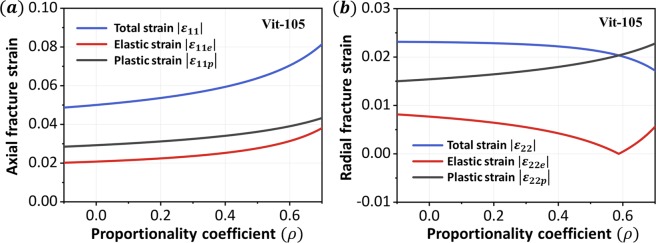
Theoretical results of fracture strain (Vit-105). (**a**) Variations in the axial fracture strain |*ε*_11_|, |*ε*_11*e*_|, and |*ε*_11*p*_| with respect to the stress proportionality *ρ*. (**b**) Variations in the radial fracture strain |*ε*_22_|, |*ε*_22*e*_|, and |*ε*_22*p*_| with respect to the stress proportionality *ρ*.

**Figure 4:**
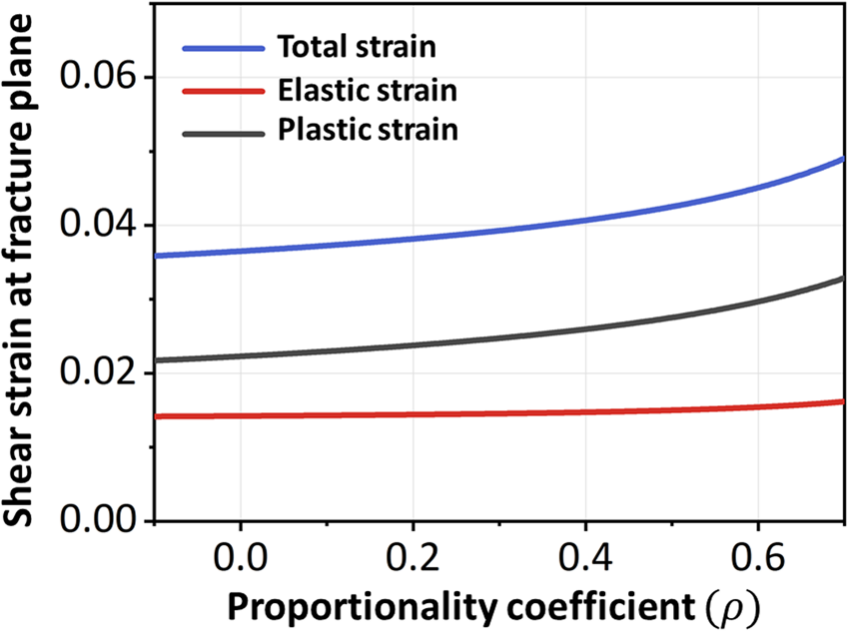
Theoretical results of plastic fracture strain along the fracture plane (Vit-105).

With the constitutive relation (Eq. (7)) and the fracture criterion (Eq. (1)), the fracture behaviors of Vit-105 round bars under different loading modes can be obtained. The material constants are shown in Table 1 (additional details about the calculations are provided in the Methods section). The dependence between the fracture strain and the proportionality coefficient *ρ* is illustrated in Fig. 3. On the one hand, as *ρ* increases, both the axial elastic strain |*ε*_11*e*_| and the plastic strain |*ε*_11*p*_|continually increase. On the other hand, due to the Poisson effect, the radial elastic strain |*ε*_22*e*_| is a tensile strain when *ρ* is small, and it will continue to decrease as *ρ* increases, eventually becoming a compressive strain. The variation in radial plastic strain |*ε*_22*p*_| with respect to *ρ* is also shown in Fig. 3(b), which exhibits an increasing trend. The shear strain along the fracture plane versus the proportionality coefficient *ρ* is shown in Fig. 4. Both the elastic strain and the plastic shear strain increase with increasing *ρ*, and the latter increases faster than the former. The increase in confining pressure is equivalent to exerting a stronger constraint on the specimen; therefore, the specimen requires more deformation to fracture.

**Table 1:** Basic data of Young’s modulus *E*, Poisson’s ratio *ν*, pure shear strength *τ*_0_, fracture angle *θ*_*uni*_, axial fracture strain *ε*_1*uni*_ and strength *T*_*uni*_ under uniaxial compressive loading for Vit-105^31,38^.

Metallic glasses		Uniaxial compression					
		Strength *Tuni* (GPa)		Fracture angle *θ*_*uni*_ (°)	Axial fracture strain *ε*_1*uni*_(%)		Radial fracture strain *ε*_2*uni*_(%)
Zr_52.5_Cu_17.9_Ni_14.6_Al_10_Ti_5_ (Vit-105)		−1.843		43	−5		2.31*
**Young’s modulus** ***E*** **(GPa)**	**Poisson’s ratio** ***ν***	**Pure shear strength τ**_0_ **(GPa)**	**Pressure-sensitivity parameter**				**Material constant** *β*
			**Fracture criterion** ***α***_***C***0_			**Yield criterion** *α*	
88.6	0.37	0.842	0.13*			0.03*	3.23*

The data with *, including the radial fracture strain *ε*_2*uni*_, material constant *β*, and the pressure-sensitivity parameters *α*_*C*0_ and *α*, are calculated by these basic data.

### Comparison of the theoretical and experimental results of Vit-1 BMG

**Figure 5:**
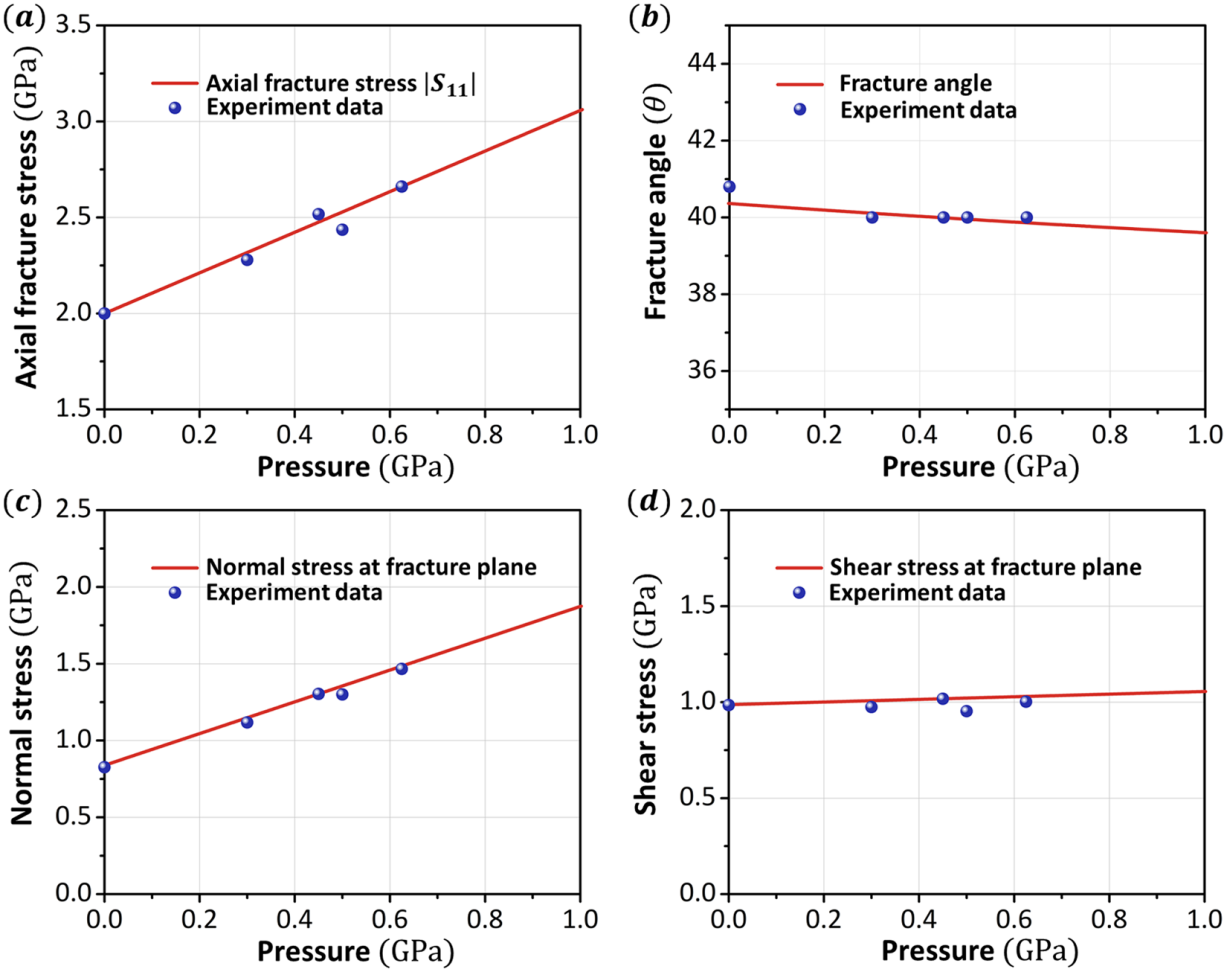
Analysis results vs. experimental data (Vit-1). (**a**) Variations in the axial fracture stress with respect to the confining pressure. (**b**) Variations in the fracture angle with respect to the confining pressure. (**c**) Variations in the normal stress on the fracture plane with respect to the confining pressure. (**d**) Shear stress on the fracture plane with respect to the confining pressure.

The complex compressive loading experiments for another typical Vit-1 BMG were reported in the literature^16,26^. The round bars were 12.7 mm in length and 6.35 ± 0.02 mm in diameter and were subject to quasistatic compression with a preapplied superimposed pressure. The loading method in these experiments is somewhat different from the proportional loading discussed in this paper. However, due to the elastic-perfectly plastic behavior of BMGs, the nominal yielding stress can be determined directly once the material yields. The preapplied hydrostatic pressure does not cause material yielding, and the constitutive relationship in the elastic deformation of the material is independent of the loading path. Thus, the experimental results can be compared directly with the case of proportional loading. The material constants are shown in Table 2, and more experimental data and calculations are shown in the Methods section. The theoretical and experimental results of Vit-1 are in good agreement, as shown in Fig. 5. Specifically, the axial stress and the normal stress on the fracture plane increase significantly with increasing pressure, whereas the fracture angle and the shear stress on the fracture plane remain approximately constant the scope of study.

**Table 2:** Basic data of Young’s modulus *E*, Poisson’s ratio *ν*^5^, material constant *β*, and pressure-sensitivity parameters *α*_*C*0_ and *α* for Vit-1 BMGs, as calculated in this work (the calculation is shown in the Methods section).

Metallic glasses	Pressure-sensitivity parameter	
	Fracture criterion *α*_*C*0_	Yield criterion *α*
Zr_41.2_Ti_13.8_Cu_12.5_Ni_10_Be_22.4_	0.3038	0.0819
**Material constant** ***β***	**Young modulus** ***E*** **(GPa)**	**Poisson ratio** *ν*
1.642	101	0.35

## Discussion

**Figure 6:**
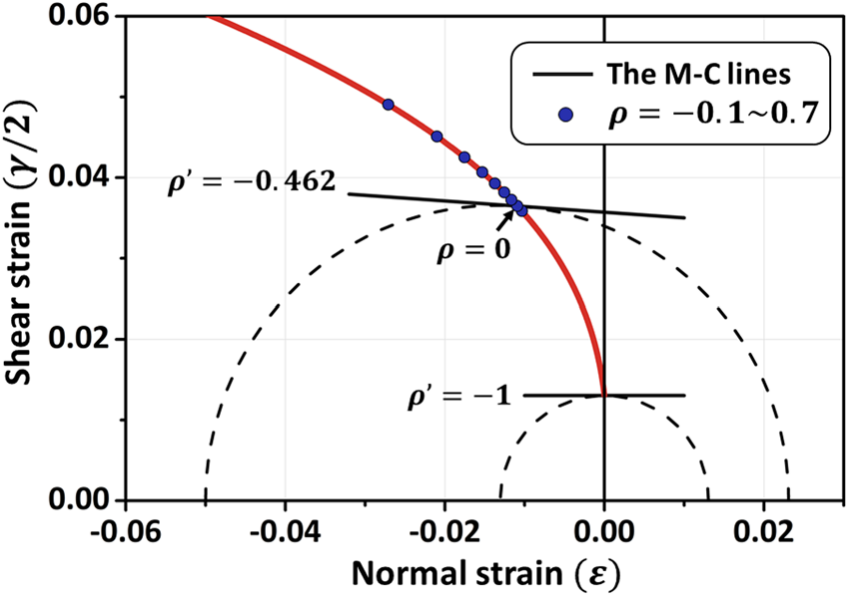
Critical fracture lines under different deformation and loading modes (Vit-105). Variation in the shear strain and the normal strain along the fracture plane (red line). The blue points represent the normal and shear strains under different loading modes (*ρ* = −0.1, …, 0.7).

The above results clearly describe the fracture behaviors of BMG round bar specimens under different loading modes. Actually, as a strain criterion, the present fracture criterion can directly predict the fracture behaviors of different deformation states. By substituting Eq. (5) into Eq. (1), the axial fracture strain of the round bar with different deformation modes (*ρ*′) can be obtained, which can be expressed as

8 $${\varepsilon }_{1}=\frac{{\gamma }_{C0}[1/(1+\rho ^{\prime} )+\beta ]\,\sin \,({\rm{2}}\theta )}{\cos \,({\rm{2}}\theta )-\chi },$$

where *χ* = (1 − *ρ*′)/(1 + *ρ*′). The radial strain *ε*_3_ can be given by the relation *ε*_3_ = *ρ*′*ε*_1_. With the fracture strain and the fracture angle, the fracture strain state along the fracture plane can be derived, and the dependence between the normal strain and the shear strain is illustrated by the red line in Fig. 6. Furthermore, eight blue points are also marked on the red line, which represent the fracture deformation states of the different loading modes (*ρ* = −0.1, …, 0.7). The Mohr’s circles and the M-C fracture lines shown in Fig. 6 correspond to cases where the material is subjected to uniaxial compression and pure shear loading, respectively. The fracture angle can be obtained from the slope of the M-C lines. The M-C lines in Fig. 6 indicate that the fracture angle tends to decrease with increasing pressure. When the fracture angle is reduced, the shear strain along the fracture plane becomes larger to reduce the inhibiting effect of confining pressure on the material failure. When *ρ* is small, we also note that the distribution of blue points is denser. In this case, the effect of the loading mode on fracture is not obvious. This result can qualitatively explain why the change in the fracture angle is not obvious in the uniaxial compression experiments with a preapplied superimposed pressure (the maximum value of *ρ* is 0.23)^16^.

**Figure 7:**
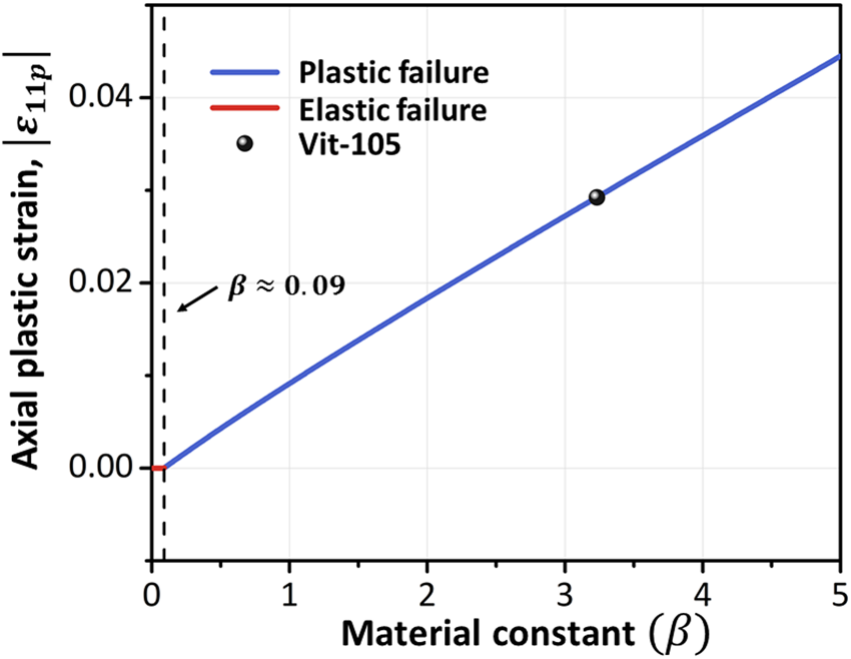
Relationship between the axial plastic fracture strain |***ε***_11***p***_| and the material constant ***β***. The elastic failure (red line) changes to the plastic failure (blue line) as *β* increases.

The analysis above shows that the predictions of the generalized M-C strain criterion strongly depend on the material constants *α*_*C*_ and *β* and the deformation state coefficient *ρ*′. Though *ρ*′ is defined as *ρ*′ = *ε*_3_/*ε*_1_ to study the fracture behaviors of the round bar in this work, this definition can also be extended to more general cases. The deformation state of materials can always be given by the three principal strains *ε*_*I*_ > *ε*_*II*_ > *ε*_*III*_, and the maximum shear strain is determined only by the first principal stain *ε*_*I*_ and the third principal strain *ε*_*III*_. Thus, the definition of *ρ*′ in general cases can be expressed as *ρ*′ = *ε*_*I*_/*ε*_*III*_. Moreover, the parameter *α*_*C*_ is the intrinsic parameter reflecting the effect of material pressure sensitivity^25^, which is closely related to the fracture angle. In addition to *α*_*C*_, it is also necessary to deepen the understanding of the meaning of *β*. We adopt the basic parameters of Vit-105 and then change *β* to obtain different results, as shown in Fig. 7. The results show that elastic fracture will change to plastic fracture as *β* increases, which indicates that the parameter *β* is related to the intrinsic plasticity of materials. Usually, for low values of *β*, BMGs tend to exhibit brittle fractures with limited macroscopic plasticity, such as La-based BMGs^32,35^. However, some newly developed BMGs with higher values of *β* can be made by adjusting the component proportion of BMGs or introducing a second crystalline phase into liquid BMGs^11,12,36^, providing greater global plasticity under uniaxial compression.

## Conclusions

We proposed a new generalized M-C strain criterion to predict the fracture behaviors of BMGs under complex compressive loading. The present strain criterion accounts for both pressure sensitivity and deformation mode. To be more specific, *α* reflects the pressure sensitivity, whereas the intrinsic material constant *β* describes the plasticity of different BMGs. To validate this new criterion, we analyzed the fracture behaviors of round bar specimens and compared the theoretical results to the experimental results of typical Vit-1 BMGs. The predicted fracture strength is consistent with the experimental results. This new generalized M-C strain criterion can predict both the fracture strength and the fracture angle, which assists the engineering application of BMG materials.

## Methods

### Derivation of the constitutive relation

The constitutive relation of BMGs can be obtained by combining the yield criterion and the plastic normality rule. The normality rule gives the direction of plastic strain increment, which can be expressed as

9 $${\dot{D}}_{ij}^{p}=\lambda \frac{\partial f}{\partial {T}_{ij}}$$

This normality rule is described by the nominal stress. Note that *f* is the flow potential (Eq. (6)) and *λ* is a coefficient. By substituting the flow potential into the normality rule, we can obtain a relation between the plastic deformation rate and *T*_*ij*_ stress, which can be expressed as

10 $$\begin{array}{rcl}{\dot{D}}_{ij}^{p} & = & \lambda \frac{\partial f}{\partial {T}_{ij}}\\ f & = & {T}_{e}+\alpha {T}_{m}={T}_{e}+\alpha {\delta }_{ij}{T}_{ij}/3\\ {\dot{D}}_{ij}^{p} & = & \lambda \frac{\partial f}{\partial {T}_{ij}}=\lambda (\frac{\partial {T}_{e}}{\partial {T}_{ij}}+\frac{1}{3}\alpha {\delta }_{ij})=\lambda (\frac{\partial {T}_{e}^{2}}{2{T}_{e}\partial {T}_{ij}}+\frac{1}{3}\alpha {\delta }_{ij})\\ {\dot{D}}_{ij}^{p} & = & \lambda (\frac{{T^{\prime} }_{ij}}{2{T}_{e}}+\frac{1}{3}\alpha {\delta }_{ij})={\dot{\bar{\varepsilon }}}_{p}(\frac{{T^{\prime} }_{ij}}{2{T}_{e}}+\frac{1}{3}\alpha {\delta }_{ij}).\end{array}$$

The effective plastic strain rate can be expressed as

11 $${\dot{\bar{\varepsilon }}}_{p}=\sqrt{2{\dot{D}}_{ij}^{^{\prime} p}{\dot{D}}_{ij}^{^{\prime} p}}=\lambda \sqrt{2\frac{{T^{\prime} }_{ij}}{2{T}_{e}}\frac{{T^{\prime} }_{ij}}{2{T}_{e}}}=\lambda .$$

Similar results can be found in another paper^37^. Considering the elastic deformation, the strain rate can be written as shown in Eq. (7).

### Material constants of Vit-105 BMG

There are three important constants in the generalized M-C strain criterion: *α*_*C*0_, *γ*_*C*0_, and *β*. Note that *α*_*C*0_ and *β* can be given by the axial fracture strain *ε*_1_ and the fracture angle *θ* of one deformation mode experiment (*ρ*′), which can be formulized as

12 $${\alpha }_{C0}=\frac{1}{tg(2\theta )(1+\rho ^{\prime} )},$$

13 $$\beta =\frac{{\varepsilon }_{1}}{{\gamma }_{C0}\,\sin ({\rm{2}}\theta )}[\cos ({\rm{2}}\theta )-\chi ]-\frac{1}{(1+\rho ^{\prime} )},$$

where *χ* = (1 − *ρ*′)/(1 + *ρ*′). The pure shear strain (*γ*_*C*0_ = *τ*_0_/G = 0.0261), the fracture angle (*θ*_*uni*_ = 43°) and the uniaxial compressive strain (*ε*_1*uni*_ = −5%) can be obtained directly from the experiments^31,38^. Another important proportionality coefficient *ρ*′ can be given by calculating the radial strain, which can be obtained by the constitutive relationship (Eq. (7)) of BMGs. We assume that BMGs exhibit elastic-perfectly plastic behavior under different loading modes; therefore, the nominal stress remains unchanged once the material yields (${\dot{S}}_{11}={\dot{S}}_{33}=0$). Therefore, according to Eq. (7), the plastic strain rate is

14 $${\dot{\varepsilon }}_{11}={\dot{\bar{\varepsilon }}}_{p}(-\sqrt{3}+\alpha )/3,$$

15 $${\dot{\varepsilon }}_{33}={\dot{\bar{\varepsilon }}}_{p}(\sqrt{3}/2+\alpha )/3.$$

With *α* = 0.03, the corresponding radial strain can be obtained as *ε*_1*uni*_ = 2.31. Finally, other parameters (*α*_*C*0_ = 0.13 and *β* = 3.23) can be given by these data.

### Material constants of Vit-1 BMG

Complex compressive loading experiments for another typical Vit-1 BMG were performed in the literature^16,26^, and the results are listed in Table 3. The axial fracture stress, the radial fracture stress, and the stress proportionality coefficient *ρ* can be obtained from these basic data, and the results are listed in Table 4. There are three material constants *α*, *α*_*C*0_, and *β* that need to be determined before comparing the theoretical results with the experiments by the new criterion. The pressure-sensitivity constant *α* in the yield criterion can be obtained by two yield stresses of different loading modes. By substituting the first (uniaxial compression) and third (pressure = 450 MPa) experimental data into the yield criterion (Eq. (6)), one can obtain *α* = 0.0819 and *f*_0_ = *τ*_0_ = 1.1 *GPa*. Moreover, *α*_*C*0_ and *β* can be obtained by the same method as discussed before. In this part, we chose the fracture strain of the third experimental data (pressure = 450 MPa) to determine these material constants. The axial fracture strain *ε*_1(*p*=450*MPa*)_ = −0.0429^16^, the calculated radial fracture strain *ε*_2(*p*=450*MPa*)_ = 0.0180, and the calculated pure shear strain *γ*_*C*0_ = *τ*_0_/G = 0.0294. Finally, *α*_*C*0_ = 0.3038 and *β* = 1.6421 can be obtained.

**Table 3:** Note that C indicates compression and C + P indicates compression and pressure.

Test type	${{\boldsymbol{\sigma }}}_{{\boldsymbol{f}}}^{{\boldsymbol{b}}}$ (MPa)	Pressure (−*σ*_3_) (MPa)	Fracture angle (degrees)	*σ*_*n*_ (MPa)	*τ*_*n*_(MPa)
C	2000 ± 69	0.1	40.8 ± 1.4	−852.5 ± 51	988 ± 34
C + P	1979	300	40	−1118	975
C + P	2067	450	40	−1304	1018
C + P	1936	500	40	−1300	953
C + P	2036	625	40	−1466	1003

Moreover, ${\sigma }_{f}^{b}$ is the fracture stress applied by the compression machine. The actual received loading of specimen is −*σ*_1_ =${\sigma }_{f}^{b}$ + p.

**Table 4:** Axial *σ*_1_ and radial *σ*_3_ fracture stresses.

Test type	*σ*_1_ (MPa)	*σ*_3_ (MPa)	*ρ* = *σ*_3_/*σ*_1_	Fracture angle (degrees)
C	−2000 ± 69	0.1	0	40.8 ± 1.4
C + P	−2279	−300	0.1316	40
C + P	−2517	−450	0.1788	40
C + P	−2436	−500	0.2053	40
C + P	−2661	−625	0.2349	40
